# Editorial: Improving medical diagnosis in rare diseases

**DOI:** 10.3389/fgene.2022.974129

**Published:** 2022-09-06

**Authors:** Natália Duarte Linhares, Kathleen M Gorman, Alfredo Brusco

**Affiliations:** ^1^ Genuity Science, Dublin, Ireland; ^2^ Department of Neurology and Clinical Neurophysiology, Children’s Health Ireland at Temple St., Dublin, Ireland; ^3^ School of Medicine and Medical Science, University College Dublin, Dublin, Ireland; ^4^ Department of Medical Sciences, University of Turin, Turin, Italy; ^5^ Medical Genetics Unit, Città della Salute e della Scienza University Hospital, Turin, Italy

**Keywords:** Rare disease (RD), whole-exome sequencing (WES), whole-genome sequencing (WGS), next-generation sequencing (NGS), diagnosis

## Introduction

A rare disease (RD) is defined as a disorder affecting fewer than 1 in 2,000 people (European Union, 2000). Although each is rare, they cumulatively involve greater than 300 million people worldwide (Nguengang Wakap et al., 2020). There are approximately 7,000 RDs reported by the Online Mendelian Inheritance in Man (OMIM) database, and 6,172 unique RDs by Orphanet, 70% of whom have childhood onset (Amberger et al., 2015; Nguengang Wakap et al., 2020).

One of the many challenges that persons living with an RD and their families face is the search for a diagnosis. Given their rarity, large number of disease entities and heterogeneous manifestation, patients often remain undiagnosed or misdiagnosed for years. The correct diagnosis has enormous implications for the patient and their family; it offers insight into the disease cause, natural history, and prognosis. It can facilitate contact with support groups and provides families with informed genetic counselling. Moreover, it can increase the understanding of the disease pathogenesis and thus improve therapeutic strategies.

More than 70% of RDs are genetic in origin (Nguengang Wakap et al., 2020). In the last decade, next-generation sequencing (NGS) technologies such as whole-exome sequencing (WES) and whole-genome sequencing (WGS) have enabled the identification of disease-causing variants in RDs. The diagnostic yields of NGS in RDs varies (24–68%) depending on the technology employed, the disease group studied, the patient age groups, clinical indications, family structures and variant types analysed (Clark et al., 2018). Genome test interpretation and reporting represents one of the challenges to laboratories seeking to implement or maximize the diagnostic yield of NGS, and guidelines have just been published in order to address this gap (Austin-Tse et al., 2022). Even though NGS affords us a better opportunity to elucidate the genetic causes of RDs, a proportion of individuals remain undiagnosed. Therefore, within this Research Topic, we created a collection of articles demonstrating emerging approaches to facilitate genetic diagnosis and discussing the impact of identifying a genetic cause in RDs. The research topic was published in September 2020 and manuscripts were received until February 2022. It received 72 manuscripts, and 23 submissions were accepted after a rigorous and constructive reviewing process.

### Studies showing applications of NGS as a frontline test in healthcare settings to accelerate rare disease diagnosis

Time-to-diagnosis is an important metric to consider in children with RDs, especially to neonates admitted to intensive care settings. Liu et al. focus on the clinical application of WES in critically ill patients in the pediatric intensive care units; the median age of children enrolled was 10.5 months (range, 1 month to 14.8 years). Whole-exome sequencing was performed in 169 critically ill children, and approximately a quarter of them were diagnosed with a monogenic disorder. They show that WES played a key role in family decision-making and clinical treatment and may improve the prognosis of some children and reduce the economic burden on families and the society.

### Bioinformatic and molecular analysis approaches and results from genomics centers or diagnosis laboratories

Santos et al. describe a new protocol for the procedure of preconception screening of consanguineous couples by WES. They considered all genes listed in the Clinical Genomics Database as causally related to autosomal recessive (AR) diseases and analysed 39 consanguineous couples using bioinformatic filters to identify pathogenic variants that were present on the same gene in both members of the couple. In 21 couples (53.8%), they ascertained sharing of heterozygosity for at least one variant considered pathogenic for an AR disease. Once the specific pathogenic variant was identified, it became possible for the couple to undergo prenatal diagnosis or, if desired, preimplantation genetic diagnosis.

Botta et al. studied a total of 570 individuals with a clinical suspect of Myotonic dystrophy type 2 (DM2), which is an autosomal dominant multisystemic disorder caused by a (CCTG)n repeat expansion in intron 1 of *CNBP*. The DM2 locus was analysed by a combination of short-range PCR (SR-PCR), tetraplet-primed PCR (TP-PCR), long-range PCR (LR-PCR), and Sanger sequencing of *CNBP* alleles. DM2 molecular diagnosis was confirmed in 187 samples analysed (32.8%) and was mainly associated with the presence of myotonia in patients.

Mellone et al. show the utility of using a targeted NGS gene panel including 221 genes in providing molecular diagnosis as a second tier-test by detecting clinically relevant variants in 71 out of 338 (21%) patients with Neurodevelopmental Disorders (NDDs). Their findings show that this NGS panel represents a powerful and affordable clinical tool, significantly increasing the diagnostic yield in patients with different forms of NDDs in a cost- and time-effective manner.

The results of Santos et al., Botta et al. and Mellone et al. demonstrate their methods have diagnostic power, and may provide useful information and potential clinical benefits for other genomic centers offering preconception screening by WES, DM2 genetic testing, or customized NGS panels for NDD, respectively.

### Applications of methodologies to promote early genetic diagnosis

Due to advances in molecular diagnosis, individuals with a RD may even be diagnosed *in utero.* Xue et al. identified variants in *TALDO1* in a fetus with Transaldolase Deficiency, supporting the application of WES in prenatal diagnosis, and further supporting that effective postpartum treatments could improve prognosis.

The early molecular diagnosis is particularly important for treatable disorders, like Congenital adrenal hyperplasia (CAH). Tolba et al. show that Multiplex ligation-dependent probe amplification (MLPA) of *CYP21A2* may improve the diagnosis and management of missed cases with atypical CAH presentations. They analysed 112 unrelated Egyptian children with CAH and 79.5% of the patients were diagnosed within the first month of life.

Liu et al. report the clinical features of eight fetuses with pathogenic variants in *CPLANE1*, *TMEM67*, *NPHP4*, and *DYNC2H1* identified by WES analysis, expanding the prenatal clinical manifestations of ciliopathies. The fetuses showed prenatal diagnostic features including occipital encephalocele, polydactyly and polycystic kidneys.

Qiao et al. describe a prenatal case with Cornelia de Lange syndrome (CdLS) caused by a novel heterozygous synonymous *NIPBL* variant. RT-PCR confirmed that the variant affects splicing. Their study expands the mutation spectrum of *NIPBL* and shows the importance of in-depth analysis of apparent synonymous mutations in clinical diagnoses.

He et al. show that prenatal WES can improve the genetic diagnostic yield in anomalous fetuses with normal results both at karyotype and chromosomal microarray. They performed WES in 94 fetuses and identified a diagnostic genetic variant in 37 (39%). They also obtained fetal phenotypes by post-mortem examinations for terminated pregnancies, providing genotype-phenotype correlations and more precise interpretations of the genetic results.

Prenatal risk assessment of carriers of heterozygous X-linked deletions is a great challenge due to variable phenotype expression induced by X chromosome inactivation (XCI). Zhao et al. present four pedigrees with distinct pathogenic X-linked deletions larger than 1Mb, which were associated with completely skewed XCI in all female carriers. They also performed the first prenatal XCI pattern analysis in a female fetus carrier of heterozygous *PCDH19*-deletion to make risk prediction.

Hou et al. present a girl with Wiskott-Aldrich syndrome (WAS) due to a heterozygous *WAS* variant. WAS is a rare X-linked recessive immunodeficiency disorder; affected males show symptoms while females carrying a pathogenic *WAS* variant are usually asymptomatic. In the described girl, the complete inactivation of normal X-chromosome led to the dominant symptoms. This study illustrates the importance of performing in-depth molecular assays to confirm the diagnosis of symptomatic female carriers with X-linked disorders.

### Studies exploring incomplete penetrance and variable expressivity in rare disorders, and discussing genotype-phenotype correlations

With the expanded use of WES or WGS analysis, families with rare disorders with incomplete penetrance and variable expressivity are increasingly being reported, showing the importance of performing molecular analysis to facilitate the diagnosis of syndromes with heterogeneous phenotypes.

Yim et al. report a family with an incomplete presentation of Andersen-Tawil syndrome (ATS) diagnosed through WES; the proband had all classic symptom triad, while her father who also carried the same novel *KCNJ2* variant had only two symptoms and no cardiac manifestations.

Linhares et al. describe three patients with mild presentation of Arthrogryposis, renal dysfunction, and cholestasis syndrome (ARCS) diagnosed by WES, all of them carrying the same novel *VPS33B* variant. The authors raise awareness of the existence of a mild clinical picture of ARCS with prolonged survival, and they propose that molecular analysis of *VPS33B* and *VIPAS39* should be considered in patients with normal gamma-glutamyl transferase cholestasis.

Che et al. report a three-generation pedigree including eight family members with mild thrombocytopenia due to a novel heterozygous CYCS variant. There are only 4 pedigrees with non-syndromic thrombocytopenia reported in the literature, and the disease has been characterized with mild thrombocytopenia, normal platelet size and morphology, and no increased bleeding tendency.

Wen et al. report a novel *SOD1* missense variant (p.R116S) causing Rapid Progressive Familial Amyotrophic lateral sclerosis (ALS). Their study expands the list of ALS causing variants, and suggests a possible pathogenic determinant related to R116 mutant SOD1 neurotoxicity.

Xie et al. review pathogenic *COQ4* variants in patients with Primary Coenzyme Q10 Deficiency-7 (COQ10D7), a rare mitochondrial disorder. Their study provides a fundamental reference for the sub-classification of COQ10D7 and contribute to the knowledge of the pathogenesis, clinical diagnosis and prognosis of this disease, and to the development of possible interventions.

Peng et al. delineate the molecular features, clinical presentation, and aspects of the immunological phenotype of 6 paediatric patients with EBV-infection and N-linked glycosylation defect (XMEN) disease caused by novel *MAGT1* variants. They conclude that when evaluating patients with transaminase elevation, chronic EBV infection and EBV-associated lymphoproliferative disease, the possibility of XMEN should be considered in addition to isolated liver diseases.

Feng et al. characterize the phenotypic features in five families with Branchiootorenal syndrome (BOR) and branchiooto syndrome (BOS) due to novel variants in *EYA1* and *SIX1*, respectively; all cases exhibited a degree of phenotypic variability between or within families. The authors also demonstrate that cochlear implantation for auditory rehabilitation is a feasible option in some BOR/BOS patients.

Kim et al. report the first case of SHORT syndrome-related transient neonatal diabetes mellitus with insulin resistance due to a pathogenic *PIK3P1* variant. The patient exhibited three of the five characteristics in the SHORT syndrome acronym (short stature, deep set eyes, inguinal hernias), as well as the typical facial gestalt.

### Applications of bioinformatic methods to increase medical diagnosis considering repeat expansions, structural variants, mosaic variants and uniparental disomy

The diagnostic yield of NGS is expected to increase with the development of novel bioinformatics methods that could facilitate the detection of repeat expansions, structural variants, mosaic variants, uniparental disomy and disease-causing variants in non-coding regions.

Li et al. describe an 8-year-old boy with a novel homozygous *MYF5* variant due to paternal uniparental disomy (UPD) of chromosome 12. Their results re-emphasized the clinical importance of UPD analysis in case the results are inconsistent with recessive inheritance.

Li et al. show that WES could detect partial exonic deletion mutations even involving intronic sequences by copy number variants (CNVs) and breakpoint analysis. They described the case of a 10-year-old boy diagnosed with Duchenne muscular dystrophy (DMD) due to a *de novo* deletion spanning intron 50 and exon 51. The deletion was identified by WES and confirmed by Sanger sequencing and long-read WGS.

The accurate analysis of short tandem repeats is a critical aspect to define precise prognosis and transmission risk in more than 40 rare genetic diseases, including Fragile X syndrome (FXS). However, molecular diagnosis of such genetic features is traditionally challenging and incompletely achieved. Grosso et al. demonstrate that the use of indirect sequence capture (Xdrop technology) coupled to Nanopore and Illumina sequencing is an efficient approach to characterize repeat lengths and to identify Single Nucleotide Variants (SNVs) and small insertions/deletions (indels) in *FMR1*, the gene responsible of FXS.

Li et al. show the value of combining WES with low-coverage WGS for better characterization of structural rearrangements in RDs. They described an 18-year-old man with Nagashima-type palmoplantar keratoderma (NPPK) and diffuse white matter abnormalities in the brain. He presented with a mosaic heterozygous *SERPINB7* stop-gain variant inherited from the mother and a *de novo SERPINB7* exonic deletion identified via trio-based WES explaining NPPK. Copy number variant analysis of the WES data indicated an additional downstream heterozygous gross deletion of 18q22.3-q23. Furthermore, low-coverage WGS confirmed the 18q22.3-q23 deletion and additionally detected a mosaic 18q21.33-q22.3 deletion, together explaining the proband’s MRI findings.

## Conclusions and perspectives

It is evident that the applications of NGS can facilitate the diagnosis of RD patients, yet the translational gap between NGS-based genetic testing and clinical implementation remains, mainly due to a scarcity of infrastructure and trained specialized staff. Better public health policies are necessary to fill this gap and support the implementation of care pathways for people living with a RD (Castro et al., 2017; Tumiene and Graessner, 2021). On the 16 December 2021, the United Nations (UN) formally adopted the first-ever resolution on “Addressing the challenges of persons living with a rare disease and their families” (United Nations, 2021). The Resolution was adopted by consensus with the support from all 193 UN Member States of the General Assembly. The Member States are encouraged to progressively cover RD patients with safe, effective, and affordable essential medicines, diagnostics, and health technologies. The Resolution also encourages Member States to increase research support by strengthening international collaboration and the sharing of data. Consequently, new hope arises with the perspectives offered by this new UN resolution in the field of RDs.
